# Analysis of Lidar Actuator System Influence on the Quality of Dense 3D Point Cloud Obtained with SLAM

**DOI:** 10.3390/s23020721

**Published:** 2023-01-08

**Authors:** Paweł Trybała, Jarosław Szrek, Błażej Dębogórski, Bartłomiej Ziętek, Jan Blachowski, Jacek Wodecki, Radosław Zimroz

**Affiliations:** 1 Faculty of Geoengineering, Mining and Geology, Wrocław University of Science and Technology, Na Grobli 15, 50-421 Wroclaw, Poland; 2 Faculty of Mechanical Engineering, Wroclaw University of Science and Technology, Łukasiewicza 5, 50-371 Wroclaw, Poland

**Keywords:** lidar, dense point cloud, SLAM, 3D reconstruction, 3D data quality, surface density

## Abstract

Mobile mapping technologies, based on techniques such as simultaneous localization and mapping (SLAM) and surface-from-motion (SfM), are being vigorously developed both in the scientific community and in industry. They are crucial concepts for automated 3D surveying and autonomous vehicles. For various applications, rotating multiline scanners, manufactured, for example, by Velodyne and Ouster, are utilized as the main sensor of the mapping hardware system. However, their principle of operation has a substantial drawback, as their scanning pattern creates natural gaps between the scanning lines. In some models, the vertical lidar field of view can also be severely limited. To overcome these issues, more sensors could be employed, which would significantly increase the cost of the mapping system. Instead, some investigators have added a tilting or rotating motor to the lidar. Although the effectiveness of such a solution is usually clearly visible, its impact on the quality of the acquired 3D data has not yet been investigated. This paper presents an adjustable mapping system, which allows for switching between a stable, tilting or fully rotating lidar position. A simple experiment in a building corridor was performed, simulating the conditions of a mobile robot passing through a narrow tunnel: a common setting for applications, such as mining surveying or industrial facility inspection. A SLAM algorithm is utilized to create a coherent 3D point cloud of the mapped corridor for three settings of the sensor movement. The extent of improvement in the 3D data quality when using the tilting and rotating lidar, compared to keeping a stable position, is quantified. Different metrics are proposed to account for different aspects of the 3D data quality, such as completeness, density and geometry coherence. The ability of SLAM algorithms to faithfully represent selected objects appearing in the mapped scene is also examined. The results show that the fully rotating solution is optimal in terms of most of the metrics analyzed. However, the improvement observed from a horizontally mounted sensor to a tilting sensor was the most significant.

## 1. Introduction

Simultaneous localization and mapping (SLAM) represents one of the most significant computational problems for any 3D reconstruction. It can be applied to autonomous cars and robotics inspection missions to solve specific tasks, in particular map generation and obstacle detection. The most promising data source for performing these tasks is application of the lidar distance measurement method. Although many 3D-mapping lidar systems have been developed and are used in various applications, the quality and accuracy of the generated 3D point clouds and 3D geometries often remain insufficient, particularly in complex or unstable environments. However, one of the most rapidly growing fields of application for mobile mapping systems is underground mining, as the speed and possible automation of performing such measurements enables the rapid acquisition of dense 3D data for vast underground structures. For these reasons, constant improvement is needed in measurement system construction and point cloud data processing. New approaches to the analysis and methodological assessment of the 3D data quality are also required. One promising solution, improving the hardware side of the mapping system, is the design of a spinning lidar sensor with an additional module that rotates the sensor around another axis. This actuated lidar design, in which a scanner is combined with an actuation mechanism to scan a 3D volume rather than a single line, has already been used in numerous mobile robot SLAM applications [1].

Therefore, the main objective of our study was to quantitatively and qualitatively assess the influence of a well-known approach using lidar rotation on the accuracy and value of a 3D point cloud using an inspection robot in a test environment mimicking an underground mine tunnel. The paper is structured as follows: first, in Section 2.1, the state of the art is investigated. The scope of the investigation includes use of a rotating lidar in mobile or stationary mapping and point cloud accuracy and quality assessment methods, in particular, with respect to SLAM applications in underground environments. Then, in Section 2.2, the design of an adjustable mapping system, developed for this investigation, is presented. The SLAM data-processing workflow is described in Section 2.3, and our approach to assessing the quality of the results with the metrics utilized for this purpose is explained in Section 2.4. Next, in Section 2.5, the experimental setup used in this study is described. The results obtained from the experiments undertaken are first presented and analyzed in global terms in Section 3.1, and, then, considering local point cloud quality in Section 3.2. Finally, a summary of the research, as well as final thoughts and recommendations for implementing SLAM in underground environments, is presented in Section 4.

## 2. Materials and Methods

### 2.1. State of the Art

The data acquisition strategy for autonomous vehicles and inspection robots using the lidar system needs to be based on an understanding of specific environmental parameters, such as the presence of a variety of dynamic or static obstacles, ensuring the highest possible level of accuracy in the scanned data. Measurement systems with rotating lidar sensors enable the capture of dense and close-to-spherical data about the surrounding environment [2]. Thus, numerous research groups and companies have been engaged in the development of methods to increase the resolution of the mapped space with rotating 2D and/or 3D lidar systems. With respect to the academic state-of-the-art, comprehensive reviews can be found in the literature, focusing both on pure lidar SLAM [3] and on more sophisticated, fusion-based, methods [4]. Many commercial, user-oriented devices are also available on the market. An extensive, but not exhaustive, list of such solutions includes handheld scanners, such as GeoSLAM Horizon, GreenValley LiGrip, SatLab Cygnus and Leica BLK2GO, backpack solutions, such as Kaarta Stencil and Leica Pegasus, and complete mapping systems for UGVs and UAVs, such as Emesent Hovermap ST. The wide range of rapidly appearing and evolving SLAM systems on the market makes it difficult to provide a fully comprehensive list.

The design of a dual 2D/3D lidar mapping system and a six-degrees-of-freedom (DOF) interpolated odometry, where 2D lidar is used to enhance 3D solid-state lidar performance used on a ground vehicle platform, can be found in [5]. The design of a spring-mounted 3D range sensor that reduces irregular vibrations of the measurement deck is described in [6]. In [7], a method for focusing on specific regions of interest using a secondary rotation motor to receive high-density measurements of the surroundings with a mobile robot platform is presented. The authors tested how, by decreasing the secondary rotation speed in the specific region, the point density in this area could be increased. The authors of [8] developed a new system (named Art-SLAM) to perform point-cloud-based graph SLAM. The proposed system is capable of achieving real-time performance, maintaining high accuracy, and can also efficiently detect and close loops in the trajectory, using a three-phase algorithm. A similar method, focusing on the correction of a local point cloud alignment, is described in [9].

Construction of the scanning actuation mechanism can have an impact on different positions of the rotation center of the lidar mirror and construction itself; thus, sufficient calibration methods are needed. The description of an automatic calibration method for the actuated lidar can be found in [10]. An extension of the following calibration methods for multiple spinning laser scanners with the support of inertial/global navigation systems is presented in [11]. The results of the evaluation of an automated algorithm and a spinning/rolling lidar system for continuous-time trajectory estimation, taking into account inter-sample pose errors to handle data distortions, are described in [1]. The drawback of using a high-density dataset is its size; thus, compression frameworks and algorithms are needed. For example, reference [12] describes a detailed investigation of a geometry compression method created for a spinning lidar point cloud.

Point cloud accuracy and data-quality assessment are amongst the most important factors for the creation of reliable, error-free methods used in laser scanning. To tackle the recurrent problems of misregistration, outlier detection and over-completeness, a comparison of several methods is needed. In [13], the use of mobile indoor 3D scanning methods are described, which are applied to a dozen different scanned locations, using five commercial indoor mapping prototypes with respect to error metrics, which do not operate on a manually given proximity scale, but on different proximity scales. High-dense point cloud analyses include simplification methods, which enable significant size reduction, while retaining sufficient variability of the geometry. Well-known algorithms exist that combine incremental/hierarchy clustering or iterative simplification [14]. Surface reconstruction using a robust diagnostic algorithm for more resistant outlier detection and a technique for plane-fitting applying a minimum covariance determinant are presented in [15]. In [16], this is used as an alternative approach for the assessment of local surface damage in civil structures. In the same way, photogrammetry-based 3D mapping of road distress detection is considered for use with unmanned aerial vehicles in [17].

With regard to the underground mining environment being investigated in the present study, SLAM solutions and applications in real or artificial spaces need to be considered. Underground workings are characterized by significant irregularities in the surrounding geometries and by dustiness that can affect the performance of the mobile system and the quality of measurements obtained. The results presented in the studies referred to below confirm that SLAM-enabled laser scanning represents a promising method for underground mining tunnel mapping and examination. One of the first studies that indicated the potential for high-resolution 3D-mapping of an underground mine involved a cart-mounted 3D-laser-scanner setup and an automatic 3D-modeling method [18]. Similarly, reference [19] reported a method for solving the SLAM problem with six DOF for the accurate volumetric mapping of an abandoned mine.

The authors in [20] proposed and tested a graph SLAM optimization method in an underground mine laboratory based on a generalized iterative closest point (GICP). In [21], a system for continuous high-resolution mapping and exploration of underground spaces, with virtual-reality visualization capabilities that can be used in mobile robot rescue operations, was presented. The investigation described in [22] demonstrated a SLAM solution capable of accurately mapping underground mines at kilometer scales, using a spinning 2D-laser scanner and an industrial-grade inertial measurement unit mounted on a light vehicle. Finally, reference [23] analyzed the quality of SLAM-based mobile laser scanner (MLS) data for the accurate and efficient geotechnical monitoring of the underground mine environment. In addition, the applicability of real-time 3D SLAM based on normally distributed transform (NDT) and pose-graph optimization for complex underground space scenarios after disasters was examined in [24]. A broad assessment of handheld laser scanners for mine surveys and the validation of results with terrestrial laser scanners for reference data are presented in [25]. A summary of successful SLAM approaches for surveying underground environments, based on experiences from the DARPA Subterranean Challenge, is presented in [26].

The literature review highlighted that there are still gaps to be filled with respect to evaluation methods for comparing the results (i.e., 3D point clouds) produced with different SLAM systems, with respect to both the hardware and the software used. Developing such methods would assist in choosing suitable solutions for selected use-case environments, optimizing the cost of scanning, system complexity and data size and quality. This study aims to tackle this challenge.

### 2.2. Adjustable Mapping System Design

The purpose of developing an adjustable mapping system for this study was to enable additional, controlled rotation of the lidar device around its longitudinal axis. In consequence, the effective field of view (FoV) of the sensor can be gradually increased, almost up to the point of a full-spherical FoV (excluding occlusions caused by the sensor mounting and the robot). The adjustability of the proposed system is controlled by the operator, who can easily change the range of lidar rotation using control software options on the remotely connected tablet. In this research, we utilized these options to emulate three different strategies for lidar head mounting, which have been used in various commercial solutions and research prototypes. The details of the system configuration are presented in the following paragraphs.

**Figure 1:**
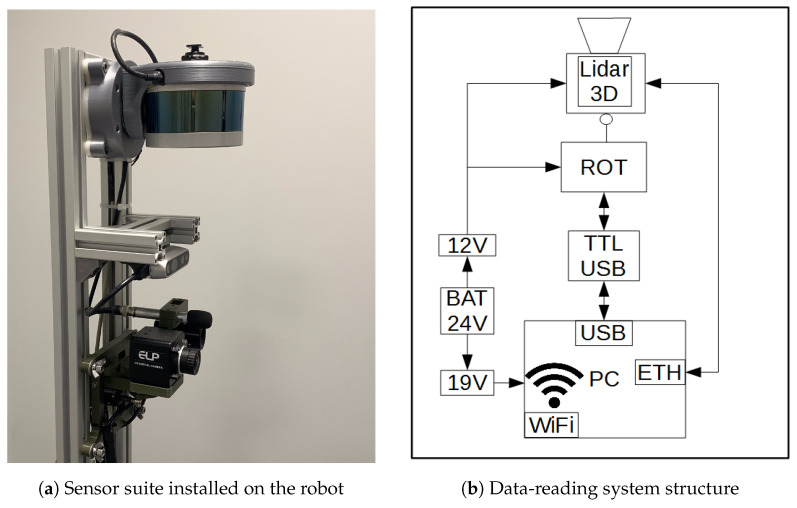
Adjustable mapping system for the mobile robot.

In this study, a 16-line Velodyne VLP-16 lidar is used. The sensor is mounted on a rotating module, mounted above the set of sensors designed for inspection purposes Figure 1a. The rotational movement is carried out using a Dynamixel AX-12A servo drive from Robotis. During movement, the desired angle range changes with a resolution of 0.29°. The supply voltage of the device is 12 V. The feedback signal of the current angular position is used to dynamically generate the rigid body transform between the lidar and the robot base reference frame. This allows the mapping to be performed in the robot frame and provides an initial estimate for transformations between consecutive lidar positions in a global frame of reference, computed by the SLAM algorithm.

**Figure 2:**
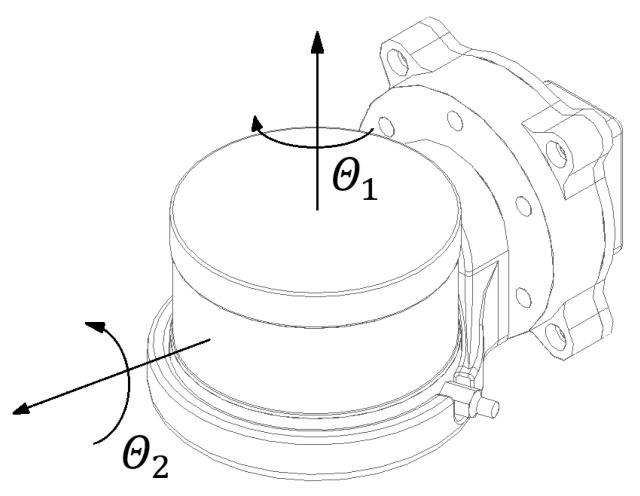
Lidar rotation module scheme. $\Theta_{1}$ represents inner laser rotation around the vertical axis and $\Theta_{2}$ denotes external rotation of the entire sensor around its longitudinal axis using an actuator.

A block diagram of the data-acquisition system with the actuator module is shown in Figure 1. Data from the lidar is sent to a computer via an Ethernet interface using a user datagram protocol (UDP). Simultaneously, the data on the current inclination angle of the actuator is transferred to the computer via the USB port of the actuator module using a half-duplex UART converter. The system is integrated with the Robot Operating System (ROS) environment and dedicated software was developed for handling the components. The software uses ROS dynamic reconfiguration parameters, which enable the setting of a stable given position of the sensor throughout the measurement session or continuous spinning of the lidar around the longitudinal axis of the robot in a given range. Due to the limitations caused by the wiring, the maximum rotation range was limited to between −90° and 90°, where the neutral horizontal position of the sensor was considered to be 0°. The control software was written using the Python language. The power supply of the system was integrated with the robot power system. Suitable voltages for powering the data acquisition computer, the lidar and the actuator module were obtained with DC/DC step-down converters, set to the appropriate output voltage. The scheme of the module is presented in Figure 2.

### 2.3. 3D Lidar SLAM

In this study, several steps of point cloud acquisition are taken to obtain coherent and noise-free global point clouds. The aim is to ensure the highest quality of results from the different methods tested so that the comparison outcomes would not be affected by external factors or imperfect execution and repetition of the experiment. The parameter values in each step are universally chosen and kept consistent for each case. Although their method-specific tuning could potentially improve the accuracy of results, it might significantly influence the data density and, thus, introduce bias into one of the most important aspects of the analysis.

**Figure 3:**
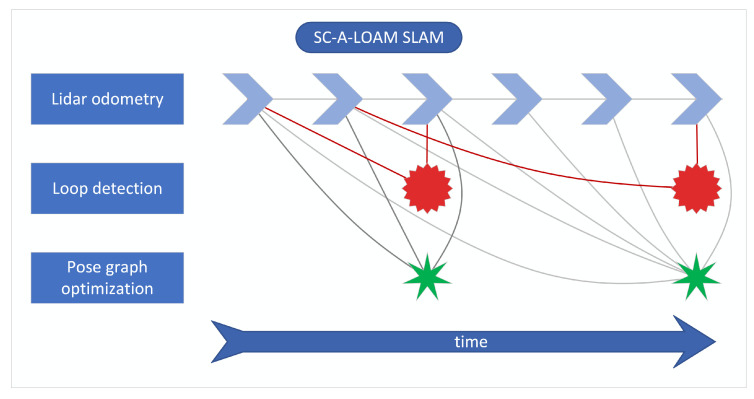
Scheme of utilized SLAM algorithm.

First, the robot trajectory is estimated in real time with the SLAM system, consisting of A-LOAM lidar odometry [27], scan context loop closure detection [28,29] and GTSAM-based pose-graph optimization [30,31,32]. A schematic overview of the method used is presented in Figure 3, where different factor graph elements are denoted as symbols with relevant connections between them. Lidar odometry, as the crucial element of the system, provides transformation estimates between the sensor reference frame and a global reference frame. The transform is calculated with a frequency of 10 Hz (equal to the lidar data acquisition frequency) based on extraction of feature points, creating edge lines and planar patches, and identifying correspondences between features found in consecutive point clouds. An initial guess is provided by the *tf* broadcaster [33], which receives the current actuator inclination angle and uses it to calculate the transform from the lidar frame to the robot base frame. The SLAM workflow is based on open-source implementation of SC-A-LOAM [28,29], with the addition of using rotation module feedback to provide an initial guess of the lidar pose transformation and to prevent distortion of the point clouds.

**Figure 4:**
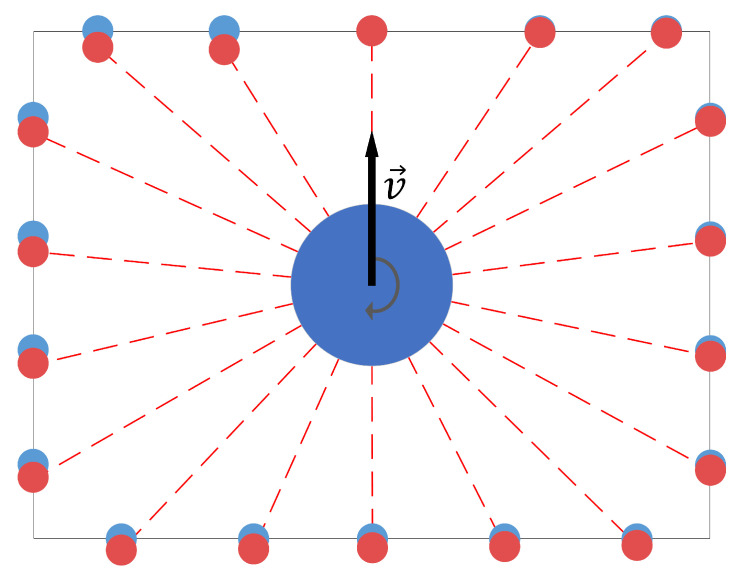
Illustration of point cloud motion distortion in a simple room seen from the top view. Raw point cloud in red; undistorted points in blue.

An important part of the applied SLAM algorithm is correction for motion distortion of point clouds. The lidar used in the study acquires 360° 3D point clouds in constant motion, revolving internally around its z-axis. One revolution (a sweep) takes 0.1 s and an aggregated set of point coordinates is sent by the sensor to the PC. However, when the scanner is in motion, the points acquired between the start and the end of the sweep have a slightly different frame of reference. This results in aggregating in each point cloud points acquired from slightly different positions, introducing a systematic error into the measurements (Figure 4). Since the individual point acquisition timestamps are known, it is possible to correct for the sensor ego-motion, provided that at least an approximate motion of the sensor during the sweep is known. Lidar odometry is utilized to reproject the points to the mutual reference frame of the point acquired at the end of the sweep.

Lidar odometry provides a quick and constant, but preliminary, sensor pose estimate. It is susceptible to long-term drift, especially with respect to orientation in the 3D space. With a rotating sensor, such as that used in the study, this could cause unacceptable mapping results, with common errors occurring, such as double walls or rotated corridors. To prevent this, loop closure detection and pose-graph optimization are included in the software system. In our test measurements, we did not have explicit loop closures in terms of returning to the same place the robot visited earlier. Therefore, Scan Context++ was used to additionally bind the trajectory after each full resolution of the actuator. The point clouds acquired at the same actuator inclination angle are/were similar due to the low speed of the robot. The resulting matches between scans are added as a constraint to the pose graph and optimized with GTSAM, reducing the drift of the odometry algorithm.

**Figure 5:**
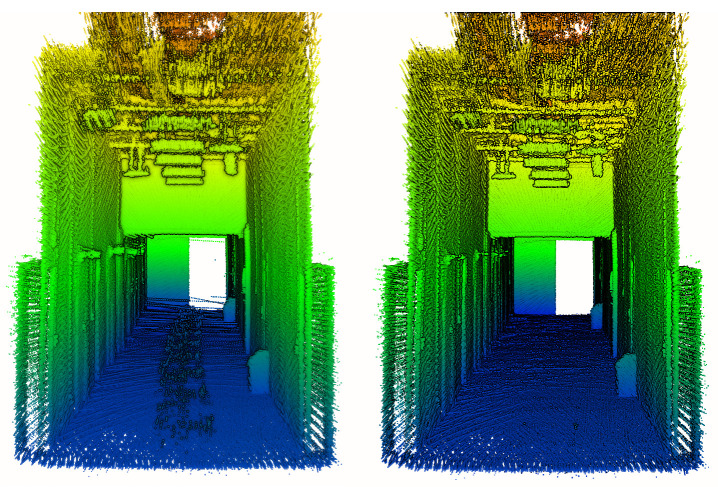
Point cloud before (**left**) and after filtering (**right**).

Then, clusters of points representing dynamic objects are removed in post-processing using removert [34]. This step enables reduction in noise of the point clouds and exclusion of moving objects accidentally appearing in the lidar field of view, such as the robot operator or the robot itself. Although the filtering procedure may slightly influence the density of the analyzed point clouds, due to the different level of robot ego-noise present in the three compared SLAM approaches, this step is necessary to allow comparison of the resulting 3D data. To minimize the influence of this step on the comparison, parameters of the filtration in all three cases were kept consistent. An example of the effect of applying removert to our data is presented in Figure 5. The noisy points are present in the central part of the scanned environment, just above the floor. They are caused by the robot elements occasionally coming into the lidar field of view. After applying the removert algorithms, this noise is successfully eliminated. In the last stage of 3D data preparation, point clouds from every scenario are cropped to the same area of interest to eliminate points that lie outside the surveyed corridor.

### 2.4. Metrics for Quality Assessment of 3D-Scene Reconstruction

Evaluating the quality of 3D point cloud data acquired with a mobile mapping system is not a straightforward task. Many metrics have been proposed and used to assess the accuracy of SLAM measurements, such as the absolute trajectory error (ATE), the relative trajectory error (RTE) and the relative position error (RPE) [35,36]. However, they focus on positioning accuracy in the global context, that is, the angular and linear drift of the algorithm in the long term, and do not convey information regarding the short-term quality of the measurements. Nevertheless, there are other important aspects of 3D-data quality, such as completeness, density and local coherence. In the context of extracting information about specific objects from the point cloud, e.g., during inspection or classification, they provide a more viable insight into the quality of geometry reconstruction [37], and, thus, constitute the centerpiece of this study.

One of the metrics that can be used to estimate the local quality of a point cloud is its density. This simply describes the number of measured points per chosen unit of reference: a volume or a surface. For this study, since the mapped area primarily contains approximately planar surfaces, surface density was calculated with respect to Equation (1) and analyzed.

(1) $$SD=\frac{N}{\Pi R^{2}}$$

where: *N*—number of neighboring points in the radius *R* around the analyzed point.

Data density provides information about the spatial distribution of points and their number, characterizing the redundancy of the measured geometry. However, this metric does not convey information on the noise level of the analyzed point cloud. To address this, another component was introduced to the analysis, namely, object reconstruction quality, that is, how accurate the measured geometry is of a single object in the scanned scene.

**Figure 6:**
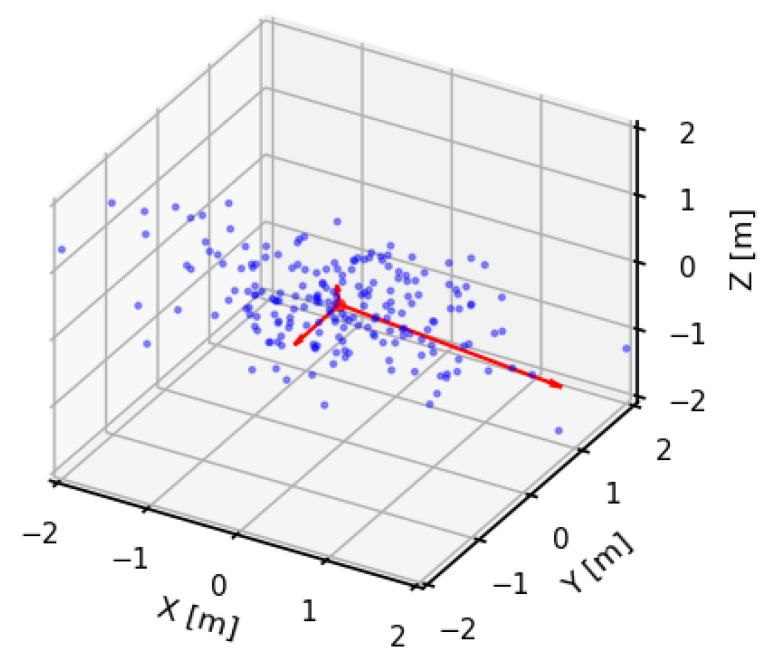
An example subset of a 3D point cloud with corresponding eigenvectors scaled by their eigenvalues.

For evaluating the object reconstruction quality, i.e., the local consistency of a point cloud, several objects were chosen as samples. They were manually identified in each point cloud and compared between different tested lidar SLAM configurations. For planar objects, such as walls, doors or floor, surface variation [38,39] (also named “change of curvature” in other reports [40]) was calculated according to Equation (2). This metric has been utilized in several investigations [15,16] to describe and identify local surface deviations on the basis of point cloud data. The metric uses local descriptors of points in the form of a covariance matrix of their neighborhood, which can be geometrically interpreted as their eigenvectors with associated eigenvalues (Figure 6). The radius, in which the neighboring points were included for calculation of the covariance matrix, was selected as 5 cm to provide detailed information, while still being above the value of the lidar ranging accuracy. Moreover, for perfectly planar objects, it was possible to additionally perform a least-square plane fit to introduce a single-number statistic for evaluation of local geometry consistency [41].

(2) $$SV=\frac{\lambda_{3}}{\sum_{i=1}^{3}\lambda_{i}}$$

where $\lambda_{1,2,3}$—eigenvalues (in descending order) of a covariance matrix calculated for the set of coordinates of neighboring points in radius R.

In the cases of two distinctive objects in the study area, namely a ceiling lamp and a trashcan, such analysis would not be meaningful since their geometry is more complex than a plane. To account for this, for all objects, point cloud resolution was additionally compared by listing the number of points per object.

**Figure 7:**
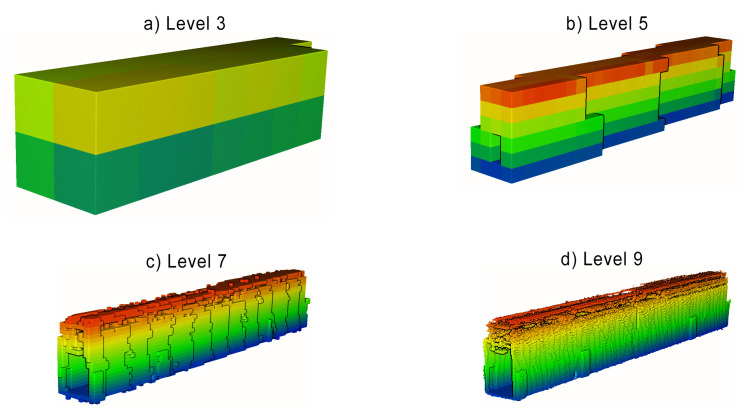
Example volumetric visualizations of an octree at levels: 3, 5, 7 and 9.

The last analyzed aspect of the point clouds acquired with SLAM is their completeness. Since the raw number of points is heavily influenced by noise and redundant measurements, other metrics need to be utilized for this purpose. Such a metric should describe a unique volume of space, containing any measured points. Two data structures commonly used in 3D-data processing have this property: a voxelgrid and an octree. Both divide a 3D space into a regular volumetric grid of boxes. For the voxelgrid, the size of a single voxel is fixed, and an octree contains a multiresolutional representation of the scene, sequentially dividing cells at each level into octants [42,43]. To assess the completeness of scanning the test environment, a number of occupied voxels and octree cells were compared between the tested approaches. Voxelgrid resolution was selected at the levels of 5, 10 and 20 cm based on the expected accuracy of the point cloud acquisition. Octree cells were counted at each of the levels from 1 to 12. An example of an octree volumetric representation is shown in Figure 7.

### 2.5. Data Acquisition Setup

**Figure 8:**
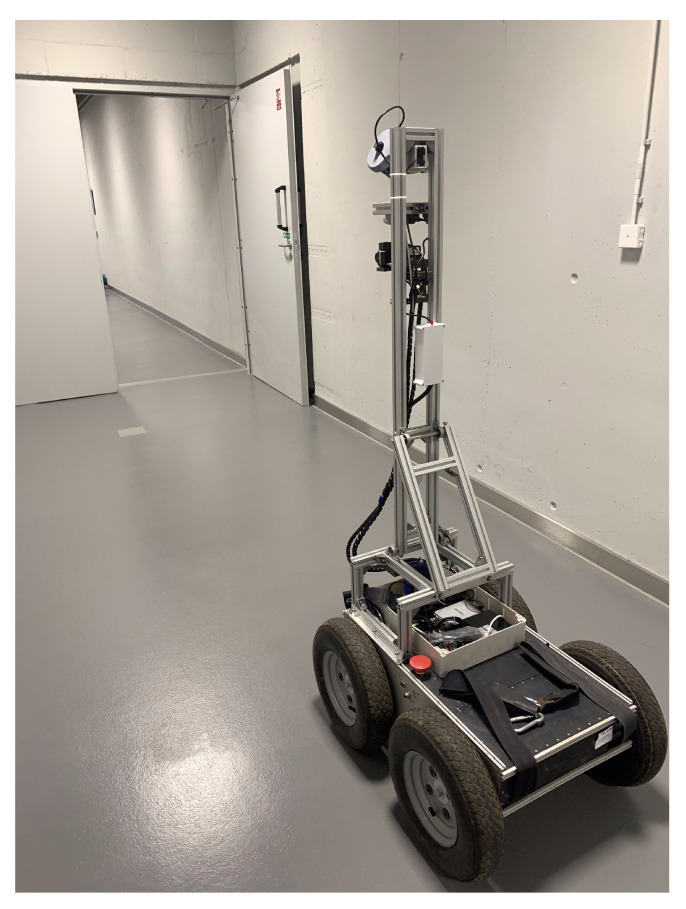
Robot during the measurements.

Several experimental data acquisitions were performed with a wheeled mobile robot (Figure 8, powered by a Robot Operating System (ROS, [44]). Each measurement scenario was carried out in the same corridor, approximately 40 m long, at the Wroclaw University of Science and Technology. The corridor contained several obstructions in front, above and on the sides of the robot, including recesses, doors and the wall above the lintel, creating occlusions for the lidar. Such conditions were chosen to simulate the problems with measurement coverage when scanning narrow linear objects, such as underground tunnels. In each case, the robot followed approximately the same straight path, through the middle of the corridor. Three common ways of utilizing the lidar sensor for SLAM were considered:

1. Sensor in fixed horizontal position, i.e., horizontal lidar;
2. Sensor rotating in the full range from −90° to 90°, i.e., rotating lidar;
3. Sensor rotating in the limited range from −45° to 45°, i.e., tilting lidar.

During the tests, the adjustable mapping system was responsible for keeping the stable position of the sensor in the first case and smoothly rotating it in the other cases. The system feedback for the inclination angle was monitored in real time to ensure that no sudden changes in the sensor inclination occurred and that the system worked correctly. Three raw measurement datasets were recorded as *.rosbag* files and later processed with the previously described SLAM workflow. Point sets were prepared for the analysis from consecutive measurements, with a horizontal, rotating, and tilting lidar containing, respectively, 1.9, 5.9 and 4.1 million points.

## 3. Results and Discussion

All the obtained point clouds were visually inspected. The topology and main dimensions of the corridor measured in the point cloud matched the ground truth, which led to the conclusion that the results of each measurement session succeeded in creating a valid 3D representation of the analyzed area. However, further analysis showed discrepancies between the results for different methods.

### 3.1. Analysis of the 3D Data Density

**Figure 9:**
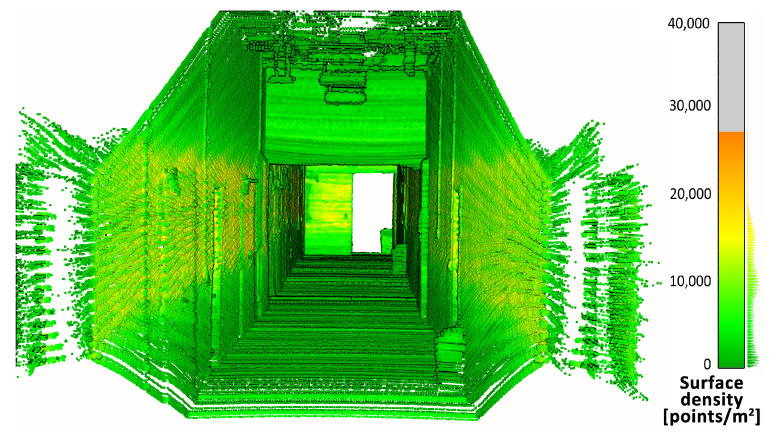
Point cloud density—horizontal lidar.

**Figure 10:**
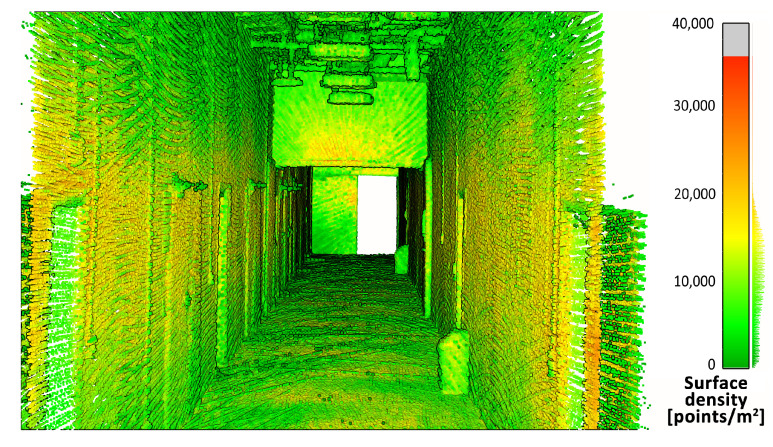
Point cloud density— rotating lidar.

**Figure 11:**
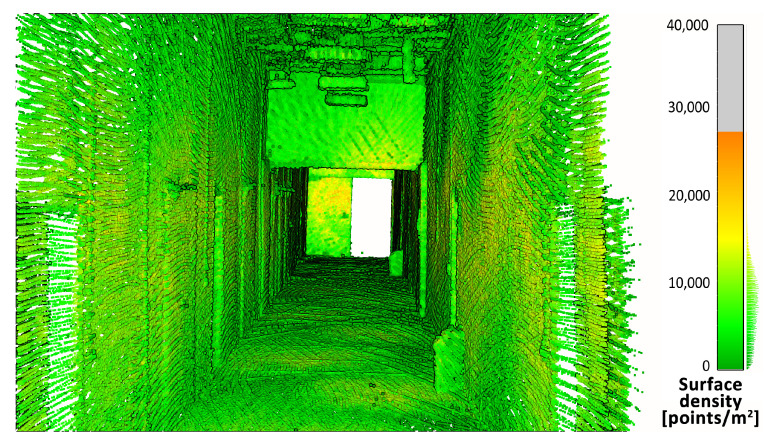
Point cloud density—tilting lidar.

**Figure 12:**
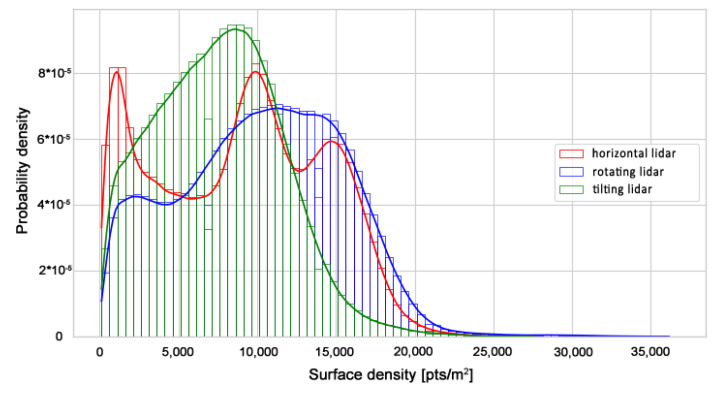
Distributions of surface densities per point.

First, the density of the point clouds was examined. For each point, a value of the density of its surroundings was calculated and represented in a 3D view with an identical color scale. The results are shown in Figure 9, Figure 10 and Figure 11. The mean densities and corresponding standard deviations were computed and are summarized in Table 1. The histograms with kernel density estimator approximations of the analyzed density values are shown in Figure 12.

In the 3D visualization of the point clouds (Figure 9), it can clearly be seen that a horizontal lidar did not provide measurements of the whole area due to its limited field of view. The most noticeable difference is located just at the starting point of the test. The density of the point cloud in this case is moderate and slightly higher at the walls at the height of the lidar during the measurements. However, examination of the histogram in Figure 12 shows its great variability. The distribution has three modes, one at a density of approximately 1000 points per m${}^{2}$ (while the mean is roughly equal to 9000 points per m${}^{2}$), which indicates that there are areas with significantly lower data coverage. This phenomenon is not observed in the distributions of the datasets from a rotating lidar or a tilting lidar. These are much smoother and exhibit a left skew towards higher density values. The 3D views in Figure 10 and Figure 11 of point clouds acquired with a rotating sensor and a tilting sensor also indicate good measurement coverage, although the former has a density higher by almost 35%, as illustrated in Table 1.

**Figure 13:**
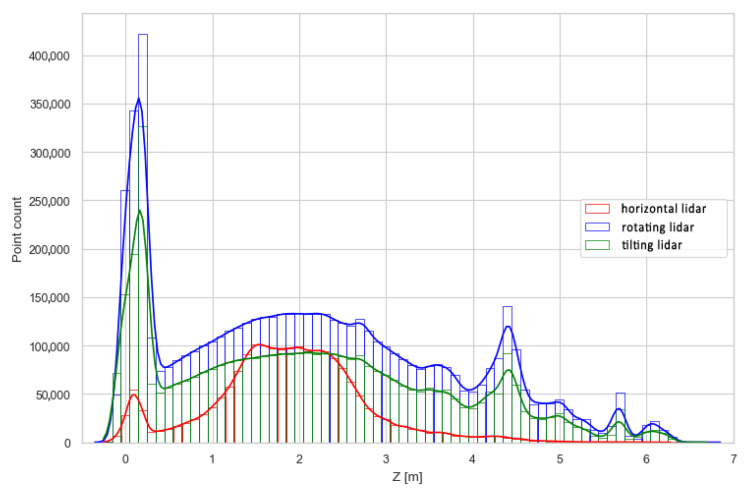
Distribution of points along the *z*-axis—absolute values.

**Figure 14:**
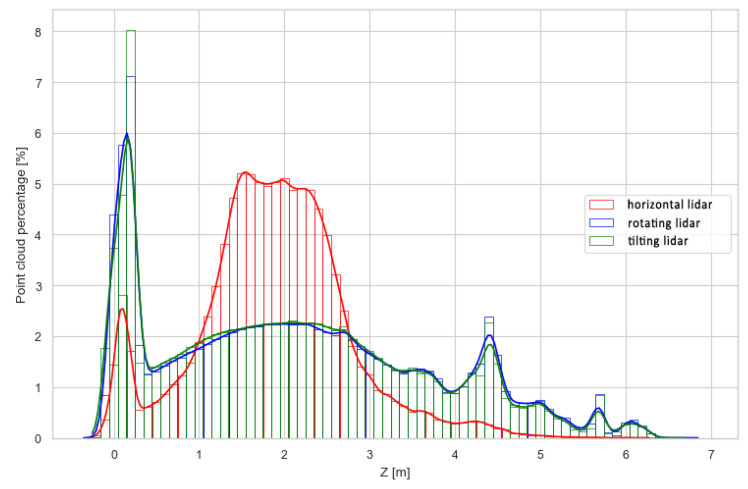
Distribution of points along the *z*-axis—relative values.

To further investigate the differences in the density of the point clouds, histograms of the points’ z-coordinates were plotted using absolute and relative values. They are shown in Figure 13 and Figure 14. While Figure 13 can be used to directly compare results from different methods to answer the questions such as, “Which method will generate the most dense point cloud and with how large deviations?”, Figure 14 is better suited to describe the internal properties of each method, i.e., “How well does the examined method represent different areas, such as the floor, walls and ceiling?”. The former question is important in terms of selecting the measurement method for a specific use case, while the latter can be utilized to set expectations and plan measurements with an already selected method, e.g., due to hardware limitations.

The graph in Figure 13 generally indicates lower data density of the horizontal lidar measurements at every height compared to the density of the other point clouds. However, from 1.5 to 2.5 m above the ground level, i.e., at the level of the sensor mounting point, the density is similar to the other methods examined for utilizing the lidar for SLAM. Another mode of distribution is located at the ground level, representing good coverage of the floor area. This peak is not present at the higher elevation, implying weak coverage of the ceiling with the measurements. These issues are further exaggerated in Figure 14, which highlights the limitation of the lidar placed horizontally on the robot. In a narrow, high corridor, this method resulted in an unevenly dense point cloud, where areas of the floor and walls at the level of the sensor position were overrepresented in comparison to areas not well-covered, such as the ceiling. The other two methods, while differing in absolute values (Figure 13), are characterized by very similar distributions of relative point counts per height. This indicates that both methods are suitable for measurements of confined linear spaces, similar to the test corridor, in terms of providing an evenly dense and complete point cloud.

**Figure 15:**
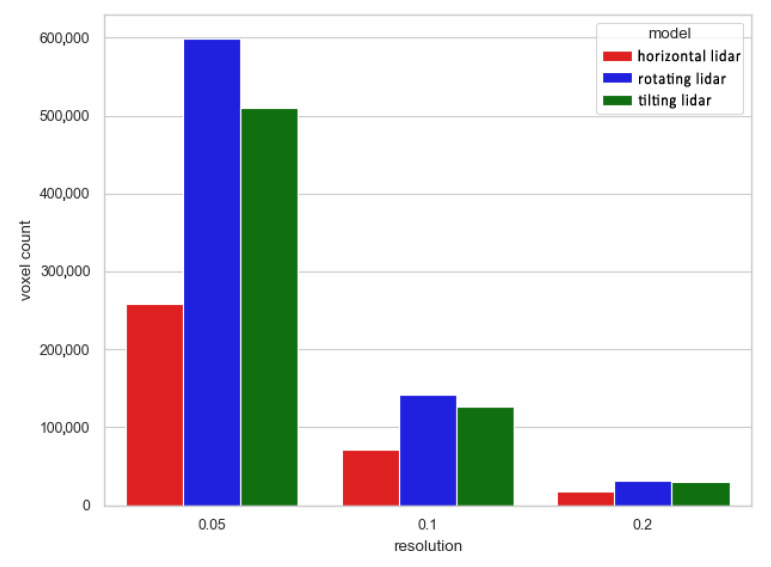
Number of voxels for different voxelgrid resolutions.

**Figure 16:**
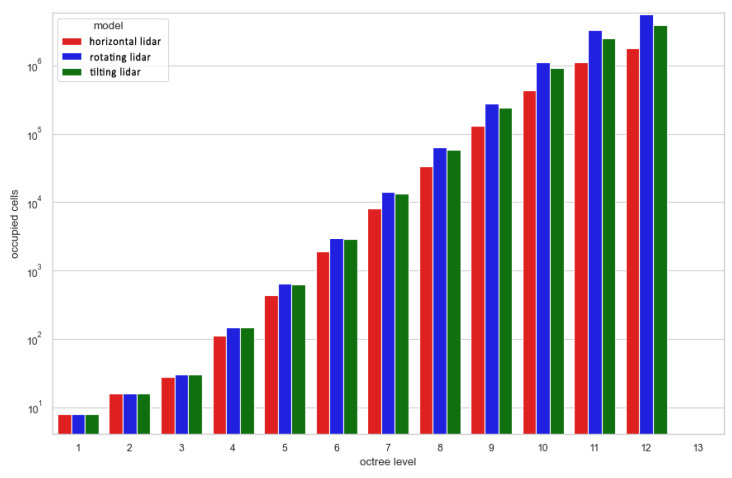
Number of occupied octree cells at each level from 1 to 12 (log scale).

A similar conclusion can be reached when analyzing point clouds downsampled by the voxelgrids and octrees, for which the number of cells occupied for each method are shown in Figure 15 and Figure 16, respectively. At low voxelgrid resolutions and low octree levels, all the point clouds contain a similar number of points, although the dataset acquired with a rotating sensor is always the most numerous, followed by the tilting and horizontal lidar methods. However, the higher the resolution and the octree level, the more visible the difference between each method. At the highest analyzed resolution, the difference between the rotating and tilting lidars also becomes significant: the former dataset contains 17% more 5 cm voxels and 44% more occupied octree cells at its 12th level.

### 3.2. Local Point Cloud Quality

**Figure 17:**
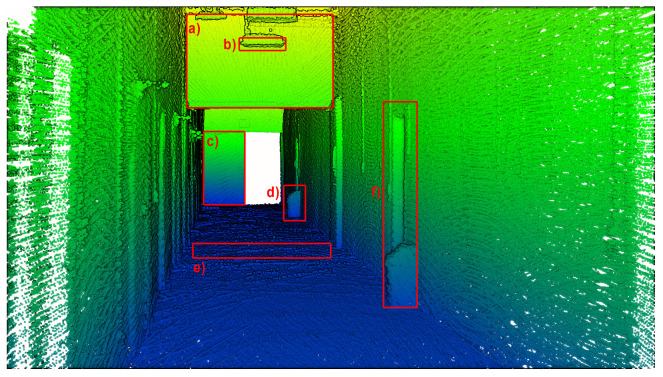
Objects selected for point cloud quality evaluation.

In the previous subsection, the three analyzed point clouds were compared in the global context, i.e., metrics were computed and examined for the whole dataset at once, describing their overall spatial distribution, completeness and density. In contrast, this subsection focuses on the examination of a few selected objects of interest located in the test area. This analysis aims to highlight the deviations in the quality of the 3D reconstruction of these objects between different methods. Six objects chosen for the detailed selective analysis are marked with red boxes, annotated from (a) to (f), in Figure 17.

**Figure 18:**
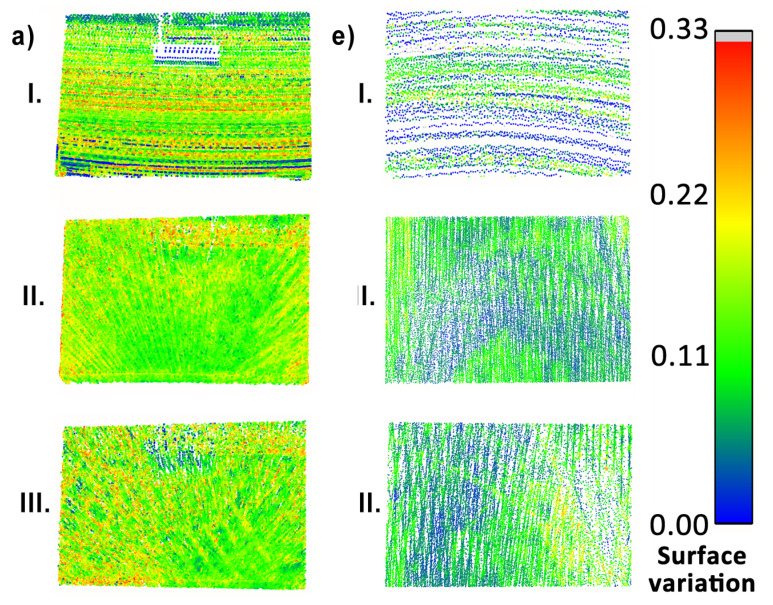
Point cloud surface variation comparison—objects (a) (**left**) and (e) (**right**).

**Figure 19:**
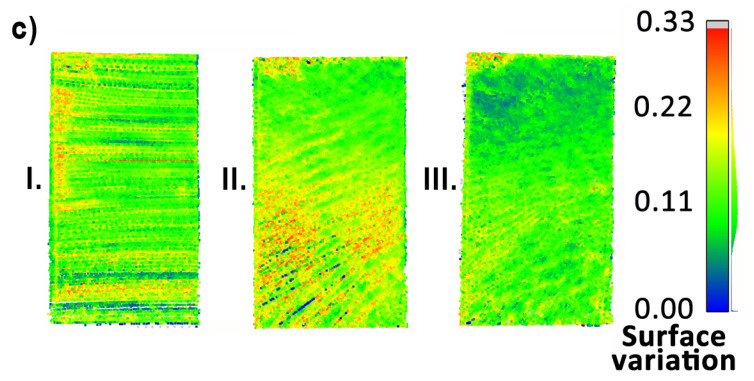
Point cloud surface variation comparison—object (c).

**Figure 20:**
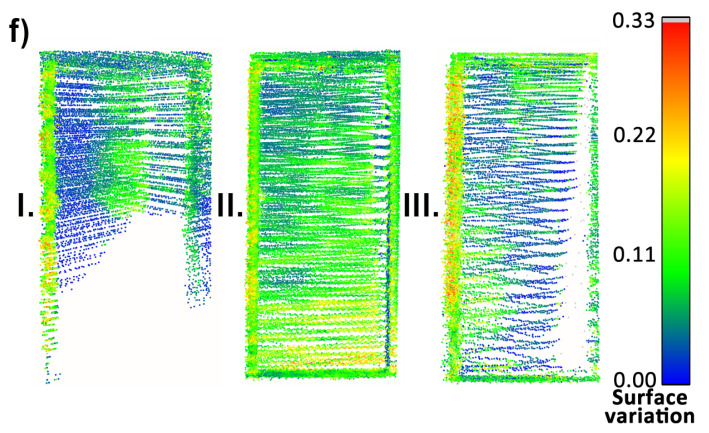
Point cloud surface variation comparison—object (f).

To begin with, the reconstruction quality of objects (a), (c), (e), and (f) was investigated, since they consist mostly of planar objects. Consecutively, they are: a vertical surface located high, a vertical surface at the same level as the measurement system, an area of the floor at the mid-section of the measurement area and a door at the side wall in the corridor. Their point clouds, colored by the calculated surface variation per point, are presented in Figure 18, Figure 19 and Figure 20. For objects (a) and (f) a huge influence of the occlusions is visible in measurements with the horizontal sensor, resulting in parts of the objects not being mapped. Surface variation values for the above-mentioned objects were similar for all tested methods. Horizontal lidar acquisition was characterized by the most extreme discrepancies of surface variation in most cases, with the areas of low values mixed with clusters of moderate and high outlier values. Rotating lidar produced point clouds that had the most coherent surface variation in cases (a), (e), and (f), but the tilting sensor achieved the best results in the case of object (c).

Local accuracy of the final point clouds was assessed using a least-squares plane fit for objects (a), (c) and (e). The results are listed in Table 2. The most consistent method was the rotating lidar, which achieved the maximum standard deviation of plane fit residuals of 36 mm, which is not much greater than the sensor ranging accuracy (30 mm). Although the residuals for horizontal and tilting lidar were also at an acceptable level, each method achieved a roughly 50% increase in the residuals’ standard deviation compared to the rotating lidar (for objects (a) and (c)). A noteworthy observation is the high compliance of the results for object (e)—visible in all parts of the floor.

**Figure 21:**
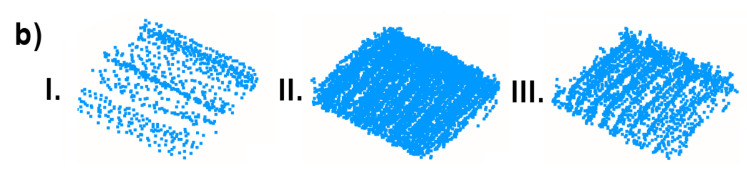
Point cloud resolution comparison for object (b)—isometric views.

**Figure 22:**
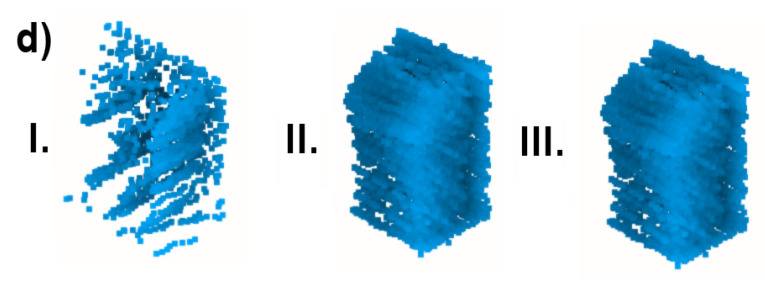
Point cloud resolution comparison for object (d)—isometric views.

Objects (b) and (d), which had more complex geometries, were analyzed only in a simplified context. Their point clouds are shown in Figure 21 and Figure 22, respectively. In the visualizations, the differences between the completeness of the 3D object reconstruction using various methods are clear: the horizontal lidar did not acquire dense and complete point clouds of those objects, while the rotating and tilting lidars successfully provided sufficient 3D data to represent a complete object. For object (b), the difference between the rotating and the tilting sensors is more noticeable than for object (d), which is caused by the unfavorable placement of object (b) for the tilting sensor in the tested configuration (i.e., tilting of the sensor did not direct its field of view much to the ceiling).

The completeness of 3D data in the context of object reconstruction, as well as the point cloud density, can be additionally summed up with a simple metric—the point count per object. Such an overview is presented in Table 3. Analyzing this metric, it can be seen that the rotating lidar acquired the most points in all cases. Compared to the horizontal lidar, it obtained from 40% more points up to seven times more points in the cases of the complex objects (b) and (d). Compared to the tilt sensor, the rotating lidar acquired roughly 50% more data, but, for object (b), the discrepancy increased to 150% more points in favor of the rotating sensor.

## 4. Conclusions

Different aspects of the 3D-data quality of three common hardware solutions utilizing a 3D lidar scanner for the SLAM problem were investigated. Multi-metric comparison was conducted to analyze factors such as local surface density and variation, plane reconstruction accuracy and numbers of octree cells, voxels and points per mapped object. This analysis enabled us to obtain insights into the behavior of SLAM in tunnel-like conditions, especially with respect to key aspects of inspection and mapping robotic missions in constrained, underground environments. Similarly to the method described in [13], in the future, our approach could be extended by performing multi-scale, multi-metric analysis of the presented metrics, using software components with use of the ROS operating system and the hardware setup described. This would enrich the results, especially when carrying out such a comparison for scenes of greater scale.

Increasing the complexity of the system through introduction of an actuator to rotate the spinning lidar around another axis greatly increased the data density and completeness, and did not negatively impact the point cloud local coherence. Although the sensor rotating in its full range generally obtained the best results, a tilting sensor achieved results that were not much worse and provided significant improvement over the static, horizontal placement of the lidar. Depending on the metrics analyzed, generally, the performance of the rotating lidar was from 35% to almost 50% better than that of the tilting lidar. The tilting lidar obtained a smoother data density distribution and almost 200% better completeness (based on voxelgrid and octree cell counts) than the horizontal lidar, while still maintaining comparable plane fit accuracy and mean data density. Choosing the right tool for the selected measurement site will depend on the dimensions of the structure, notably its height and width. The presence of objects causing numerous occlusions, common for underground mining environments, would also favor the selection of one of the actuated lidar mounts.

During inspection missions in underground mines, a massive amount of 3D data is collected with SLAM to be used for both navigation and 3D analysis. In the case of the latter, completeness of different object representations in the point cloud is crucial to enable machine learning algorithms to perform classification successfully and to correctly distinguish different objects of interest, which can then be processed with specialized, use-case-targeted algorithms. Therefore, given the results presented in Section 3.2, sensor solutions providing data denser than regular line scanners are desired. Although in this study an actuated line scanner proved to be effective, similar devices, e.g., solid-state lidars, should provide substantial improvement in data density. On the other hand, their limitations often include a reduced field of view, which may negatively impact coverage of the scanned area.

## Figures and Tables

**Table 1:** Density statistics in points per m${}^{2}$.

No	Point Cloud	Mean Surface Density	Standard Deviation
I	Horizontal lidar	8978	5249
II	Rotating lidar	10,230	5146
III	Tilting lidar	7581	3967

**Table 2:** Standard deviations of the least-squares plane fit residuals.

No	Measurement Type	Plane Fit σ [mm]		
		(a)	(c)	(e)
I	Horizontal lidar	47	28	20
II	Rotating lidar	30	36	12
III	Tilting lidar	42	52	14

**Table 3:** Comparison of the number of points per object.

No	Measurement Type	Points per Object					
		(a)	(b)	(c)	(d)	(e)	(f)
I	Horizontal lidar	38,113	836	64,347	1432	6013	23,576
II	Rotating lidar	86,618	5874	90,425	6289	44,671	38,065
III	Tilting lidar	55,151	2286	70,834	4415	25,727	20,222

## Data Availability

Data available upon request from the authors.
